# Sonocatalytic degradation of RB-5 dye using ZnO nanoparticles doped with transition metals

**DOI:** 10.1007/s11356-024-35776-4

**Published:** 2024-12-20

**Authors:** Tatiana Rodríguez-Flores, Isaías Hernández-Pérez, Gloria Elena de la Huerta-Hernández, Raúl Suárez-Parra, Catalina Haro-Pérez

**Affiliations:** 1 Departamento de Ciencias Básicas, Universidad Autónoma Metropolitana-Azcapotzalco, Av. San Pablo 420, C.P. 02128 Mexico City, Mexico; 2 Departamento de Materiales Solares, Instituto de Energías Renovables, Universidad Nacional Autónoma de México, Priv. Xochicalco S/N, C.P. 62580 Temixco, Morelos Mexico

**Keywords:** Doped-ZnO, Sonocatalysis, RB-5 azo dye, Transition metal doping, Wastewater treatment, Nanoparticles

## Abstract

In this study, ZnO was doped and co-doped with rhodium and tungsten to assess the impact of these transition metals on the sonocatalytic degradation of reactive black 5 azo dye (RB-5). Structural analysis revealed that doping ZnO with 1% Rh and W does not alter its wurtzite hexagonal structure, although minor changes in cell parameters were observed due to differences in electronic density. Interestingly, co-doping resulted in lower degradation efficiency than single doping, with W-ZnO emerging as the most effective catalyst, achieving 100% RB-5 degradation within 60 min, likely due to a higher density of oxygen vacancies and hydroxyl groups. Moreover, a 2^*k*^ factorial design identified optimal sonocatalytic conditions for W-ZnO, including a catalyst concentration of 0.75 g/L, a power tip of 225 W, and a hydrogen peroxide volume of 27 μL. The findings highlight the potential for doped ZnO nanoparticles in advanced oxidation processes and green chemistry applications, making this method an environmentally friendly alternative for wastewater treatment.

**Supplementary Information:**

The online version contains supplementary material available at 10.1007/s11356-024-35776-4.

## Introduction

Industrial activities contribute significantly to both air and water pollution. Azo-type dyes, among the major organic pollutants discharged into rivers and lakes, pose a particularly challenging issue due to their high chemical stability. Conventional methods like filtration, sedimentation, and coagulation fail to effectively remove these pollutants (Laib et al. 2022; Yaseen and Scholz 2019). Consequently, stringent global pollution control regulations have prompted researchers to focus on developing innovative and efficient water treatment technologies (Chen et al. 2020). According to recent studies, using advanced oxidation processes (AOPs) to treat polluted waters allows biodegradation management to reach a higher impurity elimination (Balaji et al. 2011; Madhavan et al. 2010a, b). The appeal of AOPs lies in their capability to degrade organic pollutants, facilitated by the generation of highly reactive hydroxyl radicals with potent oxidizing properties. Moreover, one of the most important advantages of AOPs is their ability to obtain a higher degree of mineralization (Abdelhaleem and Chu 2019; Hassaan et al. 2017; Khataee et al. 2015). Common AOPs include photocatalysis, photo-Fenton oxidation, ozonation, and sonochemical processes (Madhavan et al. 2010a, b; Rashtbari et al. 2021). Over the past decade, sonochemical degradation, or sonolysis, has gained increasing attention due to its high efficiency and ease operation in treating polluted effluents (Ghows and Entezari 2013; Sidnell et al. 2022).

In sonochemistry, the transmission of ultrasonic waves through a liquid induces cavitation, resulting in the rapid formation of nanobubbles characterized by extremely high temperature and pressure conditions, commonly referred to as “*hot spots*.” Subsequently, it undergoes swift collapse due to pressure variations, giving rise to both sonochemistry and sonoluminescence. (Suslick et al. 1986). The elevated temperatures near the bubbles cause water dissociation, generating hydroxyl radicals that effectively decompose organic compounds in the vicinity of the bubbles (Nam et al. 2003; Theerthagiri et al. 2016). However, the sonolysis process entails prolonged reaction times, leading to substantial energy consumption for the complete degradation and mineralization of organic pollutants. Therefore, to improve the degradation efficiency of organic pollutants by sonolysis processes, researchers have proposed the incorporation of catalysis (sonocatalysis) (Darvishi Cheshmeh Soltani et al. 2016). An advantage of the sonocatalytic process lies in the prevention of catalyst agglomeration by ultrasonic waves, resulting in a larger available surface area and thereby increasing the degradation of organic pollutants. Various types of catalyst like TiO_2_, ZnO, and Fe_2_O_3_ have been used for this purpose. Particularly, nanostructured catalysts are considered one of the most promising structures for removing different organic pollutants from air and water (Ahmed et al. 2013; Eskandarloo et al. 2016; Uma et al. 2020).

Different studies using ZnO to photodegrade (dyes or other organic compounds) showed that using a ratio of 1.0 g/L of catalyst decreases the total organic carbon (TOC) concentration to 70% and those organic pollutants can be easily transformed into compounds with lower toxicity under mild operating conditions (Golmohammadi et al. 2022; Rodwihok et al. 2021). At the same time, ZnO has some drawbacks, such as its limited application under visible light due to its large band gap energy and fast recombination of the generated electron (e^−^) and hole pairs (h^+^). An alternative approach to enhance ZnO’s activity involves doping it with transition metals since they can modify the band structure by inserting additional energy levels within the ZnO band gap. As a result, they promote increased charge transfer and reduce the degree of charge recombination (Paganini et al. 2018; Singh et al. 2019). In addition, the incorporation of an appropriate amount of transition metals into the ZnO structure can generate more oxygen vacancies, serving as electron traps and favoring a better electrons/hole charge separation (Khataee et al. 2020). Indeed, doping with tungsten the ZnO structure has improved its photocatalytic properties by reducing the recombination of electron–hole pairs caused by an effective charge separation (Adhikari et al. 2015). Moreover, the presence of tungsten in the ZnO arrangement also enhances its optical properties, reducing the band gap and enabling ZnO activation under visible light, a crucial aspect for photocatalytic applications (Viñes et al. 2018). Rhodium is another transition metal that can enhance the ZnO photocatalytic properties. Both rhodium and tungsten have electrons in the *d* orbitals, which are beneficial for the generation of impurities through the interaction between the *d* orbitals of the dopants and the *p* orbitals of oxygen. This interaction, in turn, has the potential to reduce the ZnO band gap energy. Additionally, the incorporation of Rh ions into the ZnO lattice creates localized levels within its gap, adversely impacting hole conductivity (Muñoz Ramo and Bristowe 2014). This way, doping ZnO with rhodium and tungsten can enhance the sonocatalytic degradation of organic pollutants. Both ions can create a synergistic effect in the ZnO electronic structure by forming new states and/or intermediate energy levels, which improve electron–hole separation and increase the generation of reactive oxygen species (ROS), which are key factors for enhancing catalytic efficiency. Additionally, Rh and W ions can improve the stability and durability of ZnO during catalytic cycles, ensuring a better long-term performance. However, despite all these advantages, to the best of our knowledge, the effect of doping or co-doping with Rh and W on the catalytic activity of ZnO for degrading organic pollutants in water through sonocatalysis has not been reported so far. Here, doped and co-doped ZnO materials with a low concentration (1.0% mol) of rhodium and tungsten are synthesized by sonochemistry. The purpose of using small quantities of Rh and W as dopants in ZnO is to have the benefits of doping while preserving the essential properties of the material, thus avoiding the introduction of unwanted defects and the formation of metallic oxides by the dopant ions. The structural, morphological, optical, and chemical properties of all synthesized materials were studied to understand their catalytic activity for degrading the RB-5 dye molecule by sonocatalysis. This innovative method opens new paths for the obtention of advanced catalysts for environmental remediation.

## Materials and methods

### Chemicals

Undoped ZnO, as well as rhodium and tungsten-doped ZnO, were synthesized using the sonochemical method. In the synthesis, zinc nitrate hexahydrate (Zn(NO_3_)_2_·6H_2_O) at 99%, rhodium chloride (RhCl_3_) at 98%, and sodium tungstate dihydrate (Na_2_WO_4_·2H_2_O) at 99% were employed as precursors. A solvent mixture of water and ethylene glycol was employed, with a four-molar sodium hydroxide solution (NaOH 4 M) serving as the precipitant agent. The dye used in the sonocatalytic test was the Reactive Black 5 (RB-5). All the chemicals were supplied by Merck and used without further purification.

### Synthesis of the undoped, Rh-doped, W-doped, and Rh-W co-doped ZnO materials

**Fig. 1:**
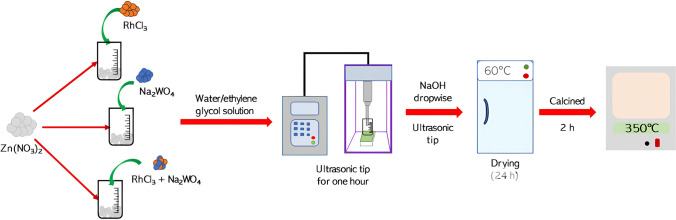
Schematic representation of the synthesis of the undoped, Rh-doped, W-doped, and co-doped ZnO by sonochemistry method

All the materials were obtained by the sonochemical method. First, 7.4 g of zinc nitrate was added to 123 mL of a water/ethylene glycol mixture (50% V/V) to obtain a molar concentration of zinc precursor of 0.2 M. Then, a stoichiometric amount of RhCl_3_ and/or Na_2_WO_4_ was added to the zinc nitrate solution to obtain 1.0% mol of Rh and W and 0.5% mol Rh-0.5% mol W, for the doped and co-doped ZnO, respectively. After that, the solutions with the precursors were sonicated by an ultrasonic tip (Ultrasonic processor FS-450N) for 1 h using a frequency of 40 kHz and a power of 135W. The next step was the addition of 20 mL of NaOH 4 M dropwise under ultrasonic irradiation until pH of 12. Finally, the white precipitate was washed several times with ethanol and water, filtered and dried at 60 °C for 24 h. Then, the resulting materials were heat-treated at 350 °C for 2 h in a static air medium. Figure 1 shows a scheme of the sonochemistry synthesis of the undoped, doped (W or Rh), and co-doped ZnO. White powders were obtained for pure ZnO and W-ZnO, while gray powders were observed for Rh-ZnO and RhW-ZnO.

### Nanoparticle characterization

The materials were morphological and structurally characterized by scanning electronic microscopy (SEM JEOL 7600F) with a 2.3 Å resolution, × 50,000 magnification, and 5.0 kV acceleration voltage. EDX mapping and spectra were performed in a TEM/STEM microscopy (JEOL ARM-200F) to illustrate the elemental distribution and percentages within the samples. To estimate the mean particle size and size distribution, the ImageJ 1.53e software was employed. XRD analysis of the samples was carried out using a Siemens X-Ray diffractometer (D5000 operating with a copper source, an accelerating voltage of 40 kV, and an emission current of 35 mA). The crystallite size was estimated applying the Scherrer equation (equation S1 in SI) to the most intense diffraction peak (101 plane). The lattice parameters were calculated by employing Bragg’s law principles and using the hexagonal wurtzite structure (equation S2 in SI), and a Rietveld refinement was also carried out to corroborate the obtained results (refer to SI for details). The UV–Vis absorbance of the catalyst was assessed within the 350–900 nm range using a Varian Cary I equipped with an integration sphere DRA-CA-30I. The optical band gap was computed from the UV–Vis absorbance spectra using Tauc’s equation and the baseline approach method; the Urbach energy was determined from the InF(*R*) vs *hν* plot (equations S3 to S6 from SI). The room temperature photoluminescence of all catalysts was examined using a fluorescence spectrophotometer (Varian Cary Eclipse) with an excitation wavelength of 370 nm, employing a xenon lamp as the excitation source. The hydrodynamic size of undoped ZnO, Rh-doped, W-doped, and RhW co-doped ZnO materials dispersed in water was determined using dynamic light scattering with a Zetasizer ZS90 (Malvern Instruments). Colloidal dispersions were prepared by mixing 1 mg of the sample in 40 mL of ultrapure water, employing an ultrasonic tip with 135 W for 8 min. To confirm the absence of organic components corresponding to the precursors and identify the functional groups of the synthesized nanoparticles, Fourier transform infrared spectroscopy (FT-IR) was conducted in fully attenuated reflectance mode across a range of 400–4000 cm^−1^ using the Perkin Elmer FT-IR spectrometer (Frontier).

### Sonocatalytic degradation

The sonocatalytic efficiency of undoped ZnO and ZnO doped and co-doped at 1.0% with rhodium and tungsten was evaluated using reactive black 5 (RB-5) azo dye as a pollutant. Sonocatalytic reactions were conducted employing a probe sonicator (Ultrasonic processor FS-450N) with a power of 270 W, and H_2_O_2_ was used as a reaction enhancer with a concentration of 6 × 10^−3^ M. The initial concentration of RB-5 azo dye was 20 ppm. All reactions were monitored until complete dye degradation, except for pure ZnO, which was followed for 5 h. To eliminate the influence of photocatalysis, all reactions were carried out in complete darkness. The monitoring was performed at 597 nm, the most representative absorption wavelength of the RB-5 azo dye. Furthermore, the sample exhibiting the highest RB-5 degradation in the shortest time was selected to study the effect of the ultrasonic probe power on dye degradation by varying it from 180 to 360 W. These experiments were monitored for 80 min. Before initiating the reactions, the dye solution with the catalyst underwent stirring in complete darkness for 1 h to establish adsorption–desorption equilibrium. In all reactions, measurements were taken every 5 min during the initial 20 min and subsequently at 20-min intervals. All samples were filtered using a Teflon filter with a pore size of 0.2 μm.

The reactions were carried out at the natural pH of the dye (pH = 6.8). Finally, the degradation efficiency (DE) was calculated using Eq. (1):

1 $$\text{DE}\%=\frac{{A}_{0}-{A}_{t}}{{A}_{0}}\times 100$$

where *A*_0_ is the initial absorbance and *A*_*t*_ is the absorbance at time *t*.

### Experimental design

To understand the sonocatalytic process and evaluate operational parameters such as catalyst dosage, ultrasonic power tip, and hydrogen peroxide concentration, a complete factorial 2^*k*^ design was used. In this investigation, the response variable was the percentage of degradation, and all experiments were replicated three times. The RB-5 degradation as a function of the operational parameters was transformed into dimensionless values (*A*, *B*, and *C*) with coded values at levels: − 1 and + 1, as outlined in Table 1.

**Table 1:** Experimental ranges and levels of the operational parameters

Parameters	Symbol	Ranges and levels	
		− 1	+ 1
Catalyst (g/L)	*A*	0.5	1
Ultrasonic probe power (W)	*B*	180	270
Hydrogen peroxide (μL)	*C*	18	36

Computational analysis, such as mathematical modeling, statistical analysis, and optimization of the process variables of the experimental data were conducted using the Minitab 19® statistical software.

## Results and discussion

### Characterization of undoped, doped, and co-doped ZnO with rhodium and tungsten

**Fig. 2:**
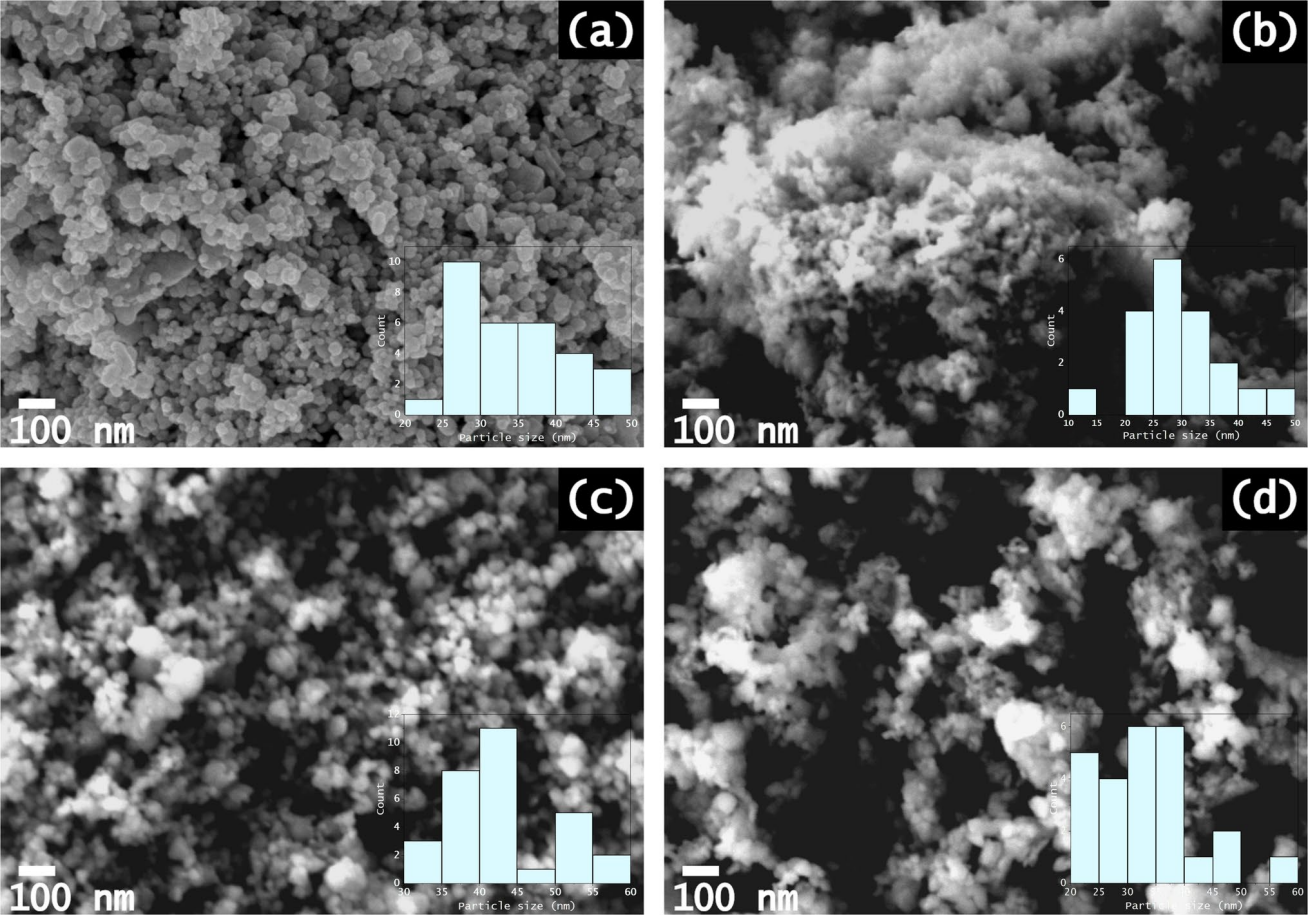
Micrographs of ZnO NP’s, with a 50,000 × magnification for (**a**) undoped ZnO, (**b**) 1.0%Rh-ZnO, (**c**) 1.0%W-ZnO, and (**d**) 1.0%RhW-ZnO

The SEM micrographs of the undoped ZnO, Rh-doped, W-doped, and Rh-W co-doped ZnO are shown in Fig. 2. In this figure, we can observe that the insertion of both rhodium and tungsten does not significantly modify the morphology of ZnO. The main morphology is almost spherical, consistent with observations reported by other authors (Akir et al. 2016; Mohamed et al. 2019). However, the spherical morphology is not that marked for Rh-ZnO, where more agglomeration is noticeable. The estimated particle size of the samples is around 35 ± 7 nm for the undoped ZnO, 30 ± 7 nm for Rh-ZnO, 43 ± 6 nm for the W-ZnO, and 34 ± 8 nm for RhW-ZnO. The presence of the dopant ions in the nanoparticles was confirmed by EDX analysis (see Fig. S1 from SI file).

**Fig. 3:**
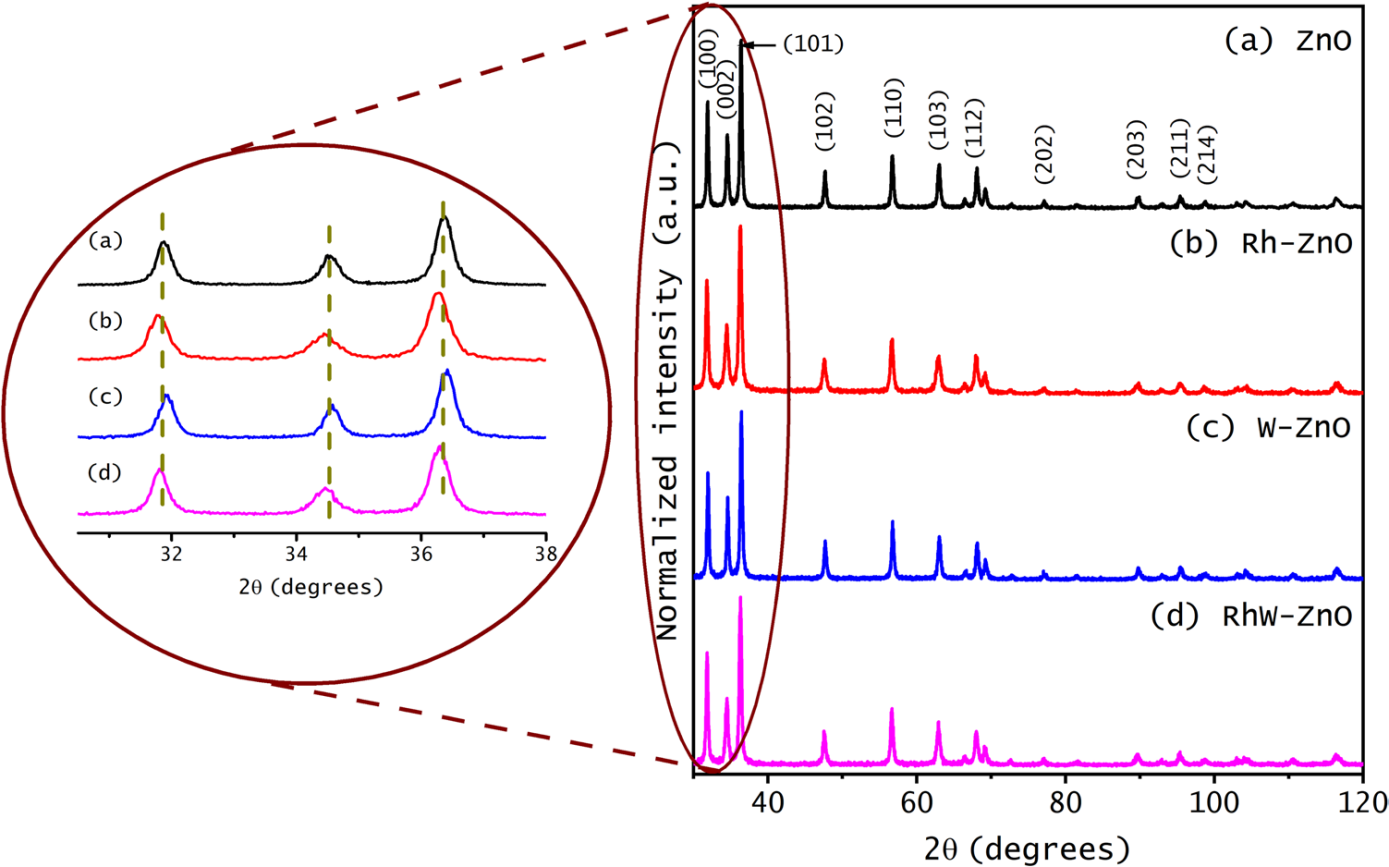
NP’s XRD patterns for (**a**) undoped ZnO, (**b**) doped 1.0%Rh-ZnO, (**c**) doped 1.0%W-ZnO, and (**d**) co-doped 0.5%Rh and 0.5%W-ZnO

The effect of dopant and co-dopant ions of rhodium and tungsten on the structure and crystallinity of ZnO was determined by X-ray powder diffraction, and the corresponding patterns are depicted in Fig. 3. In all cases, the diffraction peaks correspond to a hexagonal ZnO wurtzite structure, and additional peaks related to other metal oxides are not detected. However, rhodium insertion generated a shift towards smaller angles, while the opposite behavior was observed for W-doped samples. In the case of co-doping with rhodium and tungsten at the same percentage, there is a shift to smaller angles as reported for the Rh-ZnO sample. To analyze the changes in the diffraction peaks, structural refinements were carried out by Rietveld method, employing the FullProf Suite (5.2) software. For more details, see Table S1 and Fig. S2 (from SI). The effect of doping with rhodium and tungsten is also reflected in the cell parameters and crystallite size. As can be seen in Table 2, doping with Rh expands the cell parameter, *a*, from 3.2427 to 3.2502 Å, and the *c* cell parameter from 5.1986 to 5.2081 Å. Conversely, W doping results in a contraction of cell parameters to 3.2314 Å for *a* cell and to 5.1818 Å for *c* cell parameter. In the case of co-doping, the insertion of Rh and W ions in ZnO increases the cell parameters to 3.2463 Å and 5.2022 Å for *a* and *c*, respectively. These results are similar to those obtained from Rietveld refinement, which corroborates the insertion of the dopant ions in the lattice structure of ZnO. The observed changes can be attributed to the substitution of Zn ions with Rh or W ions, primarily due to differences in electronic density that affect lattice spacing by altering the electrostatic forces between ions. The ionic radii of Rh^3^⁺ (0.68 Å) and W⁶⁺ (0.64 Å) are smaller than that of Zn^2^⁺ (0.74 Å), leading to compressive or tensile strain within the crystal structure (Moafi et al. 2013). Furthermore, Rh can be in two oxidation states 3 + and 4 + , where the substitution with Rh^3+^ ions is more probable due to its stability. On the other hand, W has an oxidation of 4 + and 6 + ; these both ions can generate a positive charge excess imbalance leading to the formation of different defects such as oxygen vacancies or zinc interstices for charge compensation (S. G. Kumar and Rao 2015). This explains the slight increase in cell parameters when ZnO is doped with Rh and co-doped with both ions, and the subtle contraction with the insertion of W. These findings are in good agreement with existing references (Adhikari et al. 2015; Z. Chen et al. 2016; Ngom et al. 2009).

**Table 2:** Crystallite size (in nm), cell parameters (in Å), and cell volume (Å^3^) of ZnO nanoparticles: undoped ZnO, doped 1.0%Rh-ZnO, doped 1.0%W-ZnO, and co-doped 1.0%RhW-ZnO

Sample	Crystallite size (nm)	Cell parameter					
		Bragg			Rietveld refinement		
		*a* (Å)	*c* (Å)	Cell volume (Å^3^)	*a* (Å)	*c* (Å)	Cell volume (Å^3^)
ZnO	17	3.2430	5.1986	47.34	3.2450	5.1990	47.39
1.0%Rh-ZnO	17	3.2502	5.2081	47.65	3.2490	5.2001	47.53
1.0%W-ZnO	22	3.2314	5.1818	46.86	3.2300	5.1870	46.29
1.0%RhW-ZnO	20	3.2463	5.2022	47.48	3.2450	5.2014	47.43

However, concerning the crystallite sizes shown in Table 2, in the case of Rh-ZnO, the crystallite size remains unchanged with a value of 17 nm. Conversely, when ZnO is doped with tungsten and co-doped, the crystallite size experiences an increase. Specifically, in W-ZnO, the size increases from 17 to 22 nm, while in RhW-ZnO, the increment reaches up to 20 nm. This observed increase may be attributed to the stress induced by the presence of tungsten in the ZnO crystal lattice (Shunmuga Sundaram et al. 2020). The variation observed between XRD and SEM results could be due to the fact that XRD determines the crystallite sizes using statistical methods based on reflections by crystal planes, whereas, in SEM, the measurements can be affected by agglomerates of polycrystals.

**Fig. 4:**
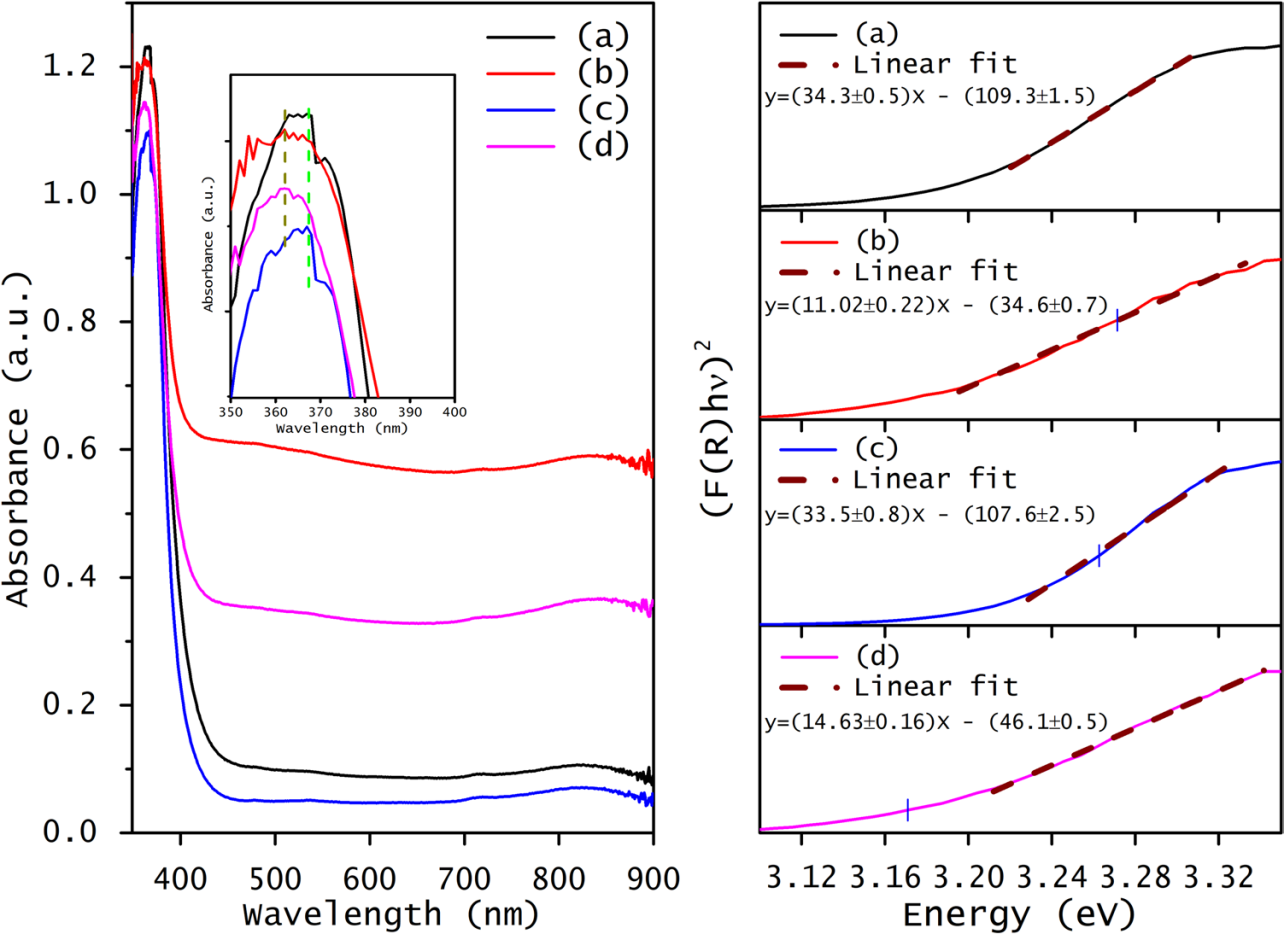
Optical absorbance spectra (left side) and (*F*(*R*)*hυ*)^2^ vs. energy (right side) for (**a**) undoped ZnO, (**b**) 1.0%Rh-ZnO, (**c**) 1.0%W-ZnO, and (**d**) 1.0%RhW-ZnO

Regarding the optical properties, all the samples display two absorption bands, one in the visible region and the other in the UV region, as illustrated in Fig. 4 (left side). In the case of samples doped with Rh (Rh-ZnO and RhW-ZnO), a subtle blue shift is observed in the UV absorption band, now located at 362 nm. The inset on the left side of Fig. 4 provides a clearer depiction of this shift in the ZnO absorption band. This observed blue shift, also documented by other researchers, is attributed to a more pronounced distortion in the ZnO lattice (Ashokkumar and Muthukumaran 2014; Rohini and Hebbar 2021). However, doping ZnO with tungsten does not alter the region of the maximum optical absorbance of ZnO, as both materials exhibit a UV absorbance band edge at 367 nm. This band is distinctive for the hexagonal wurtzite ZnO and is associated with electronic transitions. Moreover, all the materials display a slight absorption band in the near-infrared region, located at 832 nm, which is more noticeable for the Rh-ZnO and RhW-ZnO samples.

**Fig. 5:**
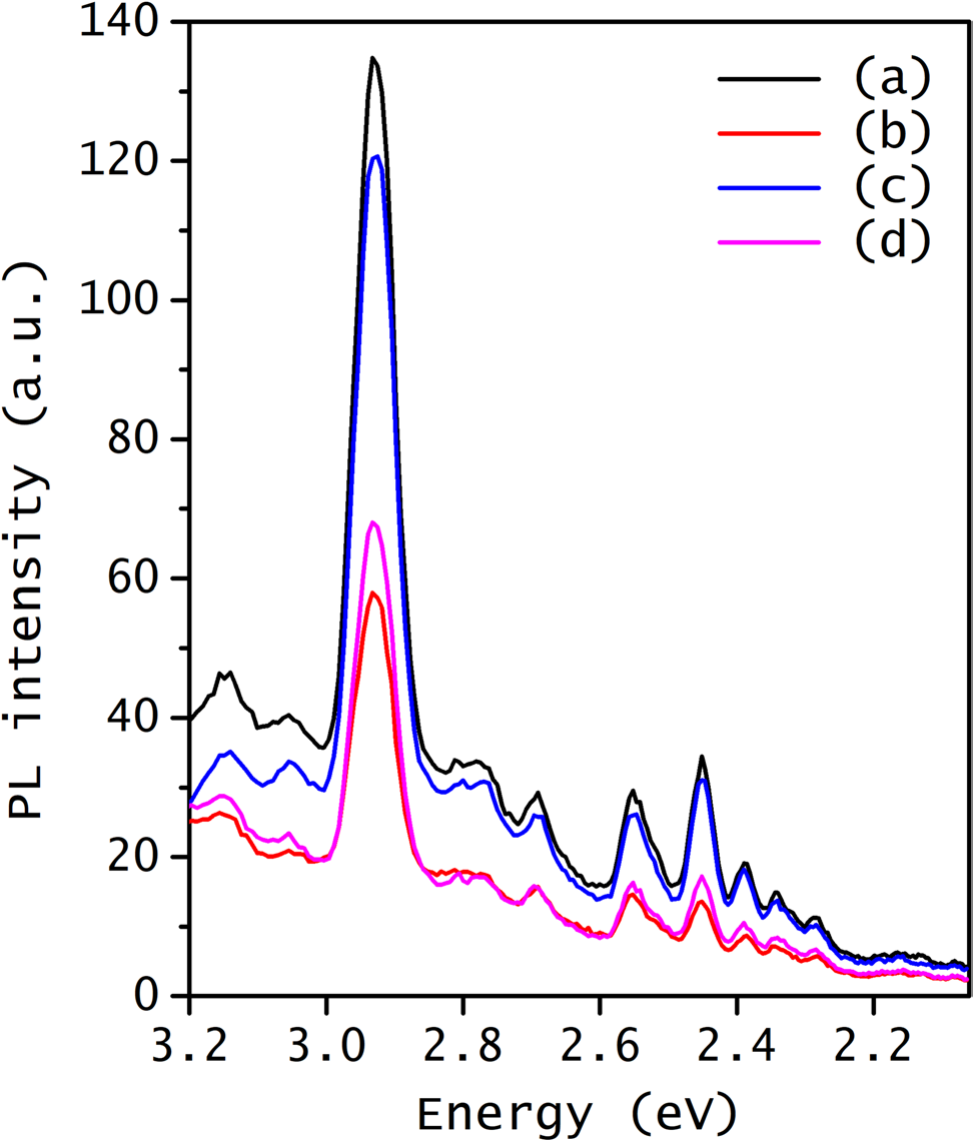
PL spectra of the undoped and doped ZnO with rhodium and tungsten: (**a**) ZnO, (**b**) 1.0%Rh-ZnO, (**c**) 1.0%W-ZnO, and (**d**) 1.0%RhW-ZnO

The optical band gap was estimated from the x-intercept of the linear equation obtained from the plot of (*F*(*R*)*hν*)^2^ versus energy, as illustrated in Fig. 4 (right side). The results indicate that rhodium doping leads to a decrease in the band gap to 3.13 eV, a value very similar to the band gap observed for ZnO co-doped with rhodium and tungsten (3.15 eV). However, doping ZnO with tungsten increases the band gap slightly from 3.19 to 3.21 eV. The band gap reduction of Rh-ZnO can be attributed to electronic transitions within the ZnO band gap due to the presence of rhodium ions. Furthermore, rhodium ions may introduce new intermediate energy levels due to the presence of different defect states or impurities formed just below the conduction band of ZnO, thus reducing the band gap energy (Russo et al. 2021). On the contrary, the rise in the band gap observed in W-ZnO could be attributed to the Burstein-Moss effect. This phenomenon explains alterations in the absorption characteristics of a semiconductor induced by doping, resulting in a change in the band gap due to band filling and an increased carrier concentration, where W^6+^ ions can create local distortions and cause repulsion between electrons in the conduction band due to the higher electronegativity, resulting in a slightly widened band gap. (Akhtar et al. 2015; Chu et al. 2014). The energy associated with defects in localized states of pure ZnO was obtained by Urbach energy (see Fig. S3 from SI). ZnO exhibited an Urbach energy of 93 meV, which is drastically modified by inserting rhodium as a dopant ion (230 meV). However, in the presence of tungsten as a dopant ion, the Urbach energy decreases up to 78 meV, and for co-doped ZnO, the obtained Urbach energy is 216 meV. The Urbach energy indicates the disorder degree within the ZnO structure generated by the presence of defects such as oxygen vacancies and interstitial zinc and localized states within the ZnO band (Ghorbali et al. 2023; Khaleel et al. 2024). The increase in the Urbach energy when ZnO is doped with Rh and co-doped with both ions is mainly attributed to the introduction of localized states and distortions within the band structure by the presence of rhodium. However, when ZnO is doped with W, the disorder degree decreases, perhaps due to a stabilization generated by the presence of W^6+^ ions which could minimize defects and distortion in the ZnO lattice structure (Janani Archana et al. 2022; Norouzzadeh et al. 2020). Crystal defects in both rhodium- and tungsten-doped as well as undoped ZnO were investigated through photoluminescence (PL) spectra, employing an excitation wavelength of 370 nm. Figure 5 shows the PL spectrum of all doped and undoped ZnO samples. In all the spectra, the same emission bands were observed but with different intensities, mainly due to the existence of different ions in the ZnO lattice. The predominant emission bands were situated in the blue-green region, and only one band was observed in the ultraviolet region. The UV emission at 3.25 eV corresponds to the recombination edge of free excitons via exciton-exciton collisions at low excitation intensity (60 W/cm^2^) and can be attributed to a radial quantum confinement effect in ZnO nanospheres (Oliveira et al. 2013). The band located at 3.14 eV can be assigned to the free-exciton emission, generated by the recombination of the formed electron–hole pairs (Amari et al. 2022; Flores et al. 2014). The variation in the intensity of this band agrees with the UV–Vis results, corroborating that W-ZnO exhibited the highest band gap, while Rh-ZnO displayed the smallest band gap. Conversely, the peaks centered between 3.05 and 2.92 eV could be attributed to the Zn vacancies, as found previously (Sahu et al. 2018).

The blue emissions at 2.77, 2.69, and 2.55 can be attributed to defect emissions caused by the incorporation of rhodium and/or tungsten ions in interstitial positions within the ZnO lattice (de Almeida et al. 2022). The emission band at 2.45 eV nm could be associated with the radiative recombination of a hole with an electron originated from an ionized oxygen vacancy (Egelhaaf and Oelkrug 1996). The green emissions at 2.34 and 2.39 eV may be associated to ionized oxygen vacancies, where their electrons recombine with a phonon hole (Lyu et al. 2002; Mekasuwandumrong et al. 2010). The presence of Zn interstitials ions and Zn/oxygen vacancies hinders the recombination process of photogenerated electron–hole pairs, leading to an increased formation of radicals that trigger degradation reactions. However, an inappropriate amount and/or arrangement of these oxygen vacancies and crystal defects can dampen degradation processes.

Based on dynamic light scattering measurements, we find that the size of the clusters dispersed in water undergoes an increase upon doping or co-doping ZnO with rhodium and tungsten, as illustrated in Fig. S4 (see SI file). Specifically, doping with Rh and co-doping with Rh and W results in an increase of the cluster size from 192 ± 11 nm for undoped ZnO to 218 ± 14 nm and 234 ± 7 nm, respectively. The most significant size increase is observed in the case of the 1.0%W doped sample, where the cluster size reaches a value of 257 ± 12 nm. Moreover, the polydispersity index indicates a relatively narrow particle size distribution when the material is dispersed in water. Additionally, all samples exhibit good colloidal stability, as it was evidenced by the hydrodynamic diameter remaining unchanged even after one month.

**Fig. 6:**
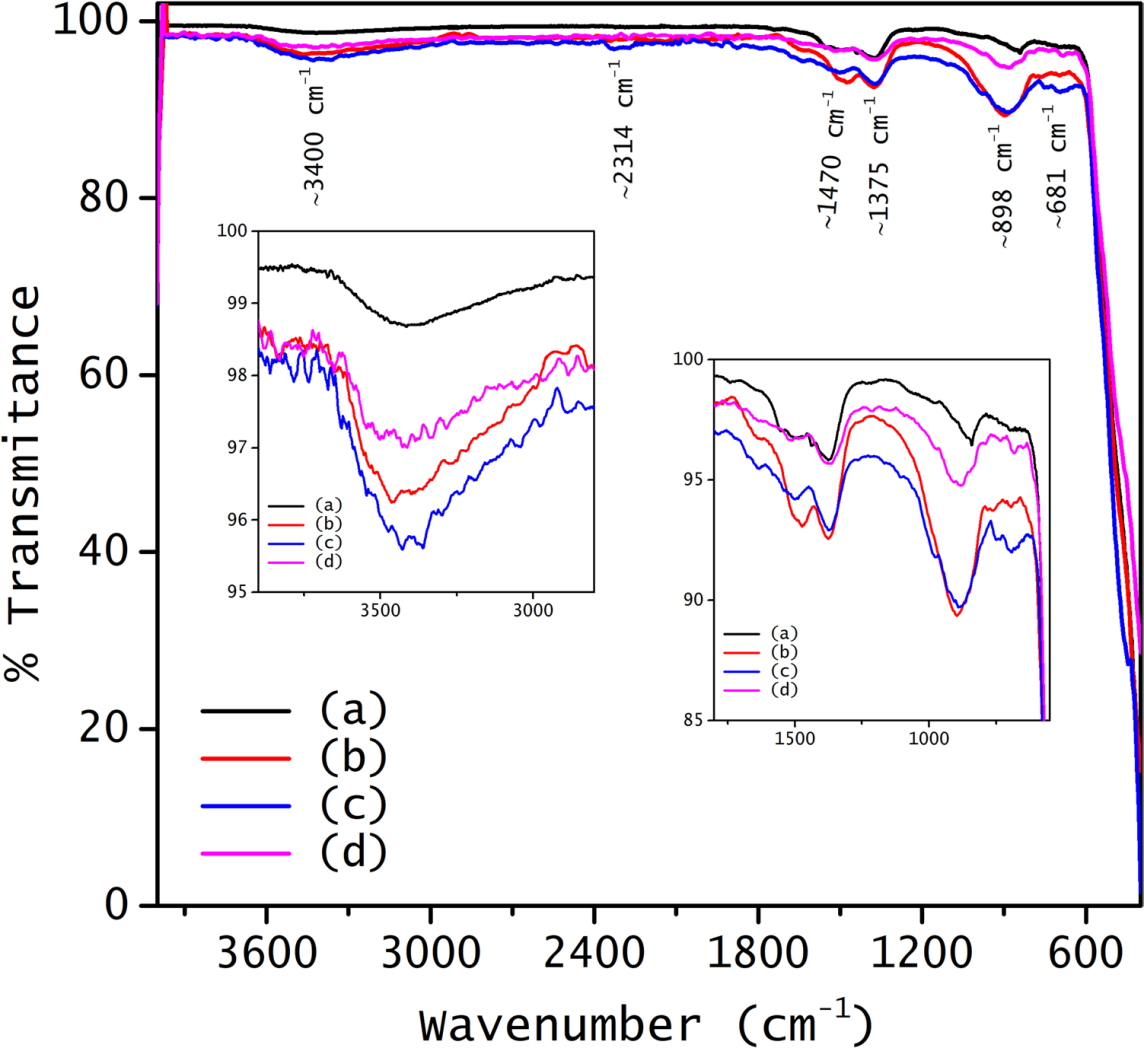
Fourier transform infrared spectra of undoped and doped ZnO: (**a**) ZnO, (**b**) 1.0%Rh-ZnO, (**c**) 1.0%W-ZnO, and (**d**) 1.0%RhW-ZnO

The presence of organic components in the samples was determined by Fourier Transform infrared spectroscopy, and the results are shown in Fig. 6. Undoped ZnO exhibits a band in the 400–650 cm^−1^ range, attributed to the Zn–O vibrations, a zoom of this region is presented in Fig. S5 (see supporting information). Absorption bands observed around 690–900 cm⁻^1^ may be associated with W–O or Rh-O. Furthermore, the two bands between 1375 and 1470 cm^−1^ are attributed to C-H bending vibrations, indicating the presence of organic residues of ethylene glycol on the surface of all samples (Mahalakshmi et al. 2020).

The band at 2314 cm^−1^ corresponds to the stretching of the CO bond, due to the CO_2_ adsorbed from the atmosphere onto the surface of the ZnO nanoparticles through M-CO type interactions, where M represents the metal, either Zn, Rh, or W (de la Cruz et al. 1990; Kaur and Singhal 2014; Mesaros et al. 2014). Finally, the broadband at 3400 cm^−1^ corresponds to the stretching vibrational mode of physisorbed O–H groups on the materials’ surface. Among the samples, W-doped ZnO exhibits the highest concentration of hydroxyl groups, followed by Rh-doped ZnO.

### Sonocatalysis evaluation

**Fig. 7:**
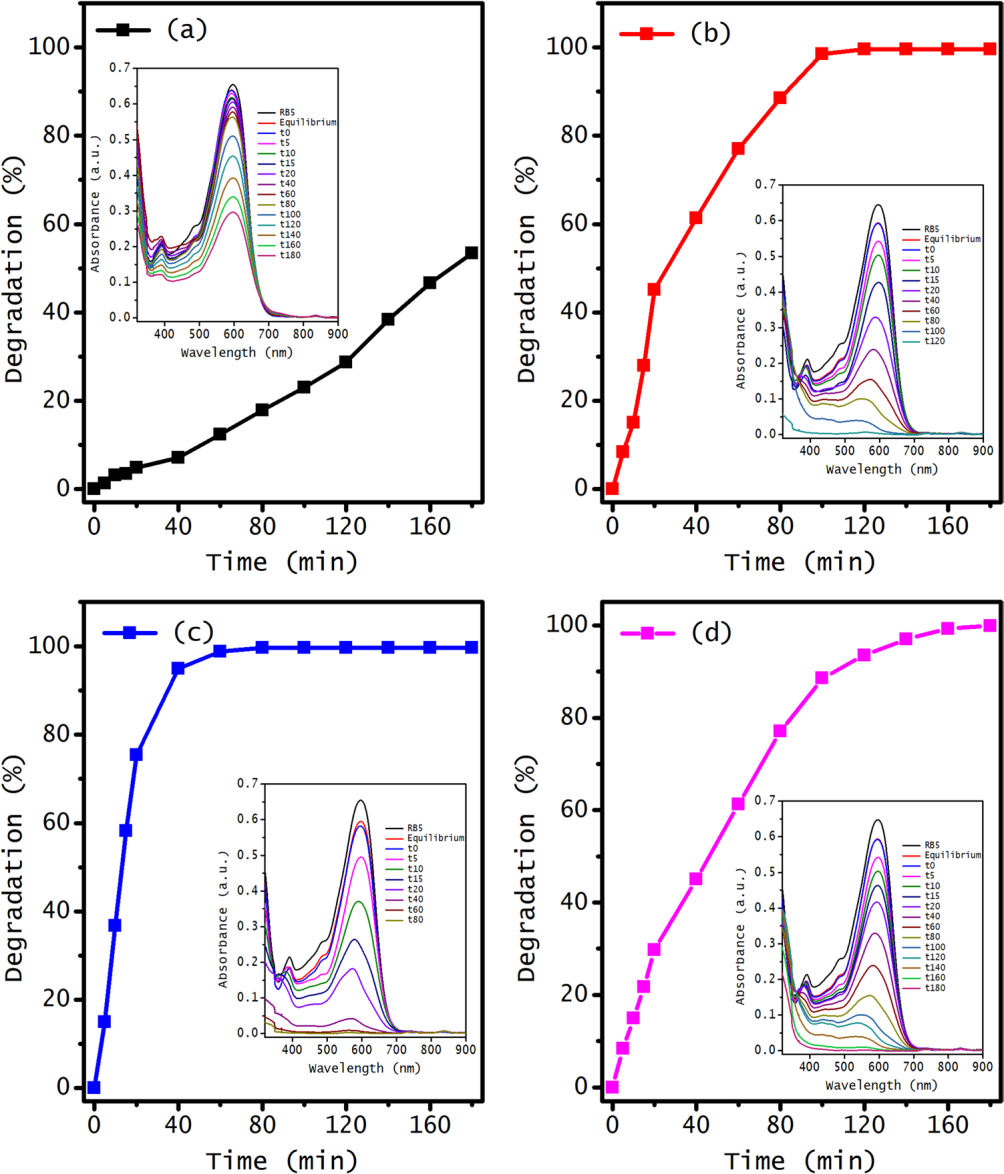
Degradation percentages of the RB-5 reactive azo dye obtained through sonocatalysis using pure ZnO, doped, and co-doped ZnO with rhodium and tungsten: (**a**) pure ZnO, (**b**) 1.0%Rh-ZnO, (**c**) 1.0%W-ZnO, and (**d**) 1.0%RhW-ZnO. The experimental conditions are as follows: a power tip of 270 W, H_2_O_2_ concentration equal to 6 × 10^−3^ M, and a catalyst dosage of 1.0 g/L. The inset shows the reduction in UV–Vis RB-5 azo dye absorbance spectra at different time intervals

The sonocatalytic reaction was conducted using a power tip operating at 270 W. The time-dependent percentages of degradation for all the materials are shown in Fig. 7. The degradation percentage is obtained from the decrease of the absorbance with exposure time, as shown in the inset of Fig. 7. The main figure highlights that doping and co-doping with rhodium and tungsten significantly enhances the degradation of the reactive black azo dye. In the case of undoped ZnO, the dye degradation reaches 53% after 180 min of reaction. However, the Rh-doped ZnO degrades the dye entirely in 120 min. Tungsten-doped ZnO exhibits the best performance, achieving complete degradation of RB-5 in approximately 60 min of reaction. Co-doping ZnO, while still effective, slows down the degradation rate compared to the doped materials, with complete degradation achieved after 180 min. Although both Rh and W individually act as electron traps that improve charge separation, their effect may differ when both are doped into ZnO simultaneously. Each dopant introduces new energy levels within the band gap, potentially leading to competition for doping sites (Alam et al. 2017). This competition can disrupt the balance of charge generation and transfer, promoting electron–hole recombination and thus reducing the formation of highly reactive species that are essential for degradation processes.

The notable RB-5 degradation observed across all doped and undoped ZnO catalysts can be attributed to the prevention of catalyst agglomeration and an increase in the available specific surface area induced by ultrasonic waves. In addition, it is possible to promote catalyst valence band electrons to the conduction band by the “*hot spots*,” since the quick collapse of vapor bubbles in the system after their formation and growth releases energy in the form of heat (Suslick et al. 1986). Hence, ultrasonic waves could accelerate the transfer of contaminants from the bulk to the catalyst surface where the degradation occurs. Besides, the enhanced sonocatalytic activity of the doped and co-doped ZnO with rhodium and tungsten can be explained by the capability of these transition metal ions to trap electrons at the ZnO conduction band, thereby preventing recombination of the electron–hole pair. The improved degradation rate observed with tungsten doping can also be attributed to the higher concentration of hydroxyl groups, as supported by FT-IR analysis. These hydroxyl groups play a crucial role in catalytic reactions by effectively trapping photogenerated holes on the catalyst surface since these groups can be transformed into hydroxyl radicals under certain conditions -such as exposure to energy, UV light, or oxidizing agents like H_2_O_2_ (Giannakoudakis et al. 2015). This process inhibits or reduces the rate of electron–hole pair recombination and generates hydroxyl radicals (OH·), thereby activating redox reactions. The proposed mechanism for converting hydroxyl groups into hydroxyl radicals in the presence of energy and H_2_O_2_ is described below. First, the collision of bubbles generates extreme temperatures and pressures, releasing sufficient energy to induce the homolytic breakdown of water molecules (see Eq. (2)). During this process, a hydrogen radical generated through cavitation collides with a hydroxyl group located either on the surface of the catalyst or the surrounding medium. This interaction causes a hydrogen transfer, converting OH^–^ into OH· (Gligorovski et al. 2015), as shown in Eq. (3):

2 $${\text{H}}_{2}{\text{O}}\to \text{ OH}\cdot + \text{H} \cdot$$

3 $${\text{2OH}}^{-}+ \text{H} \cdot \to \text{ OH}\cdot \text{+}{\text{H}}_{2}\text{O} + {e}^{-}$$

The transformation of hydroxyl groups into radicals can also be carried out through the presence of oxidizing agents such as H_2_O_2_ (Eq. (4)):

4 $${\text{H}}_{2}{{\text{O}}}_{2}\text{+}{\text{OH}}^{-}\to \text{ OH}\cdot \text{+}{\text{H}}_{2}\text{O} + {e}^{-}$$

Additionally, H_2_O_2_ can also react with a hydrogen radical generated by the cavitation-induced dissociation of water, and therefore generate more hydroxyl radicals; see Eq. (5):

5 $${\text{H}}_{2}{{\text{O}}}_{2}+ \text{H} \cdot \to {\text{OH}}\cdot \text{+}{\text{H}}_{2}{\text{O}}$$

**Fig. 8:**
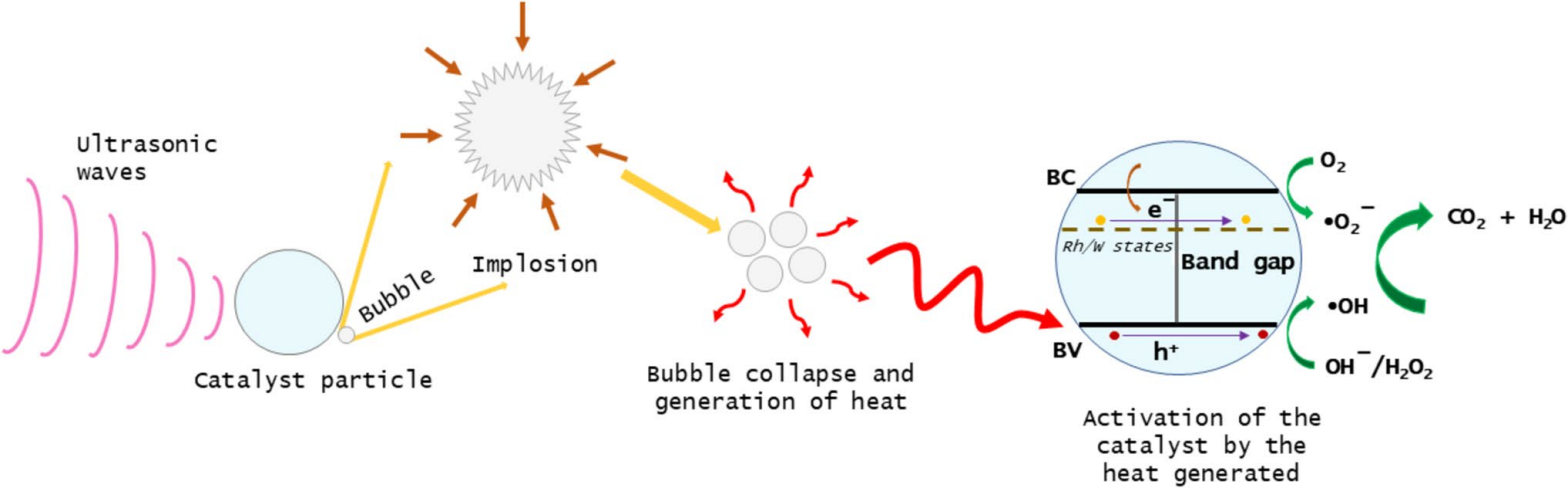
Schematic illustration of the mechanism of the degradation reactions carried out by sonocatalysis, using undoped ZnO, doped ZnO, and co-doped ZnO with rhodium and tungsten

Figure 8 provides a schematic representation of the degradation reactions facilitated by sonocatalysis. As previously discussed, the energy released during bubble collapse can activate the catalyst only if this energy equals or surpasses the band gap energy of the catalyst. Consequently, this process can induce the promotion of an electron from the valence band to the conduction band, creating a hole in the valence band. The resulting electron–hole pairs then migrate to the catalyst surface and start the redox reactions essential for the degradation of RB-5.

To investigate the impact of ultrasound power on the dye degradation process, we conducted degradation reactions at various ultrasound power levels ranging from 180 to 360 W, each lasting 80 min. These experiments (shown in Fig. S6 from SI) focused on the ZnO sample doped with 1.0% tungsten, which exhibited the fastest and most effective degradation performance. Surprisingly, the degradation percentage demonstrates a non-monotonic relationship with the power input. Initially, as the power input rises from 180 to 270 W, the degradation also increases, reaching a maximum. However, beyond this power threshold, further increases in power no longer favor dye degradation; instead, degradation diminishes as the power is raised, as observed in the curves corresponding to 315 W and 360 W (refer to Table 3).

**Table 3:** Degradation of RB-5 by sonocatalysis employing W-ZnO as a catalyst, with power variation from 180 to 360 W

Ultrasonic tip power (W)	Degradation (%)
180	89
225	95
270	100
315	99
360	93

On the one hand, the increment in the degradation of RB-5 as the power tip is increased up to 270 W may be due to the free radical’s production increase and to the higher turbulence in the solution that facilitates mass transfer (Kakavandi et al. 2019; Nuengmatcha et al. 2016). On the other hand, the reduction in sonoactivity observed at higher power levels, specifically 315 W and 360 W, could be due to a diminished contact time between the dye molecules and ZnO catalyst. Additionally, it may be associated with potential leaching of Zn ions due to increased mechanical forces and cavitation, leading to the alteration of active sites on the catalyst and a subsequent decline in its efficacy for degrading RB-5 azo dye (Bhavani and Sivasamy 2016; R. Kumar et al. 2015). This is because cavitation generates very high pressures and temperatures in a localized manner, which can alter the stability of ZnO, generating the release of metal ions during the reaction, as a result, its sonocatalytic performance decreases. This agrees with results reported by other authors (Asli and Taghizadeh 2020; Besson and Gallezot 2003; Saharan et al. 2015). Table 4 provides a summary of ZnO properties and the degradation of RB-5 azo dye.

**Table 4:** Summary of some ZnO physical properties and the RB-5 azo dye degradation

Sample	Crystallite size* (nm)	Hydrodynamic diameter (nm)	Eg (eV)	Urbach energy (meV)	Degradation** (%)
ZnO	17	192 ± 11	3.19	93	18
1.0%Rh	17	218 ± 14	3.13	230	89
1.0%W	22	257 ± 12	3.21	78	100
1.0%RhW	20	234 ± 7	3.15	216	77

^*^Crystallite size obtained by XRD in the plane (101); **degradation percentage of RB-5 at a power tip of 270 W after 80 min

As can be seen in Table 4, the sample with the lowest Urbach energy, but the highest crystallite size and hydrodynamic diameter display the highest efficiency in the RB-5 degradation. Hence, when ZnO possesses fewer defects in its lattice, sonocatalytic activity is enhanced. Therefore, the introduction of localized states by W aids in minimizing the recombination of electron–hole pairs. In addition, as illustrated in the micrographs, the W-ZnO sample exhibits the lowest agglomeration, and when combined with the increased ultrasonic tip, it increases the active sites of ZnO improving the RB-5 degradation.

### Optimization of operational conditions by 2^ k^ factorial design

To gain a deeper understanding of how factors such as catalyst dosage, hydrogen peroxide volume, and ultrasonic tip power influence the photoactivity of the 1.0% W-ZnO sample, a 2^*k*^ factorial design was employed. The applied factorial 2^3^ design comprises 8 experimental runs, as illustrated in the design matrix shown in Table 5. The corresponding degradation percentages after 80 min are also displayed in this table.

**Table 5:** Design matrix of the factorial 2^3^ design for the RB-5 azo dye degradation after 80 min

Run	Catalysts	Ultrasonic probe	H_2_O_2_	% Degradation		
1	− 1	− 1	− 1	53	51	51
2	+ 1	− 1	− 1	89	87	86
3	− 1	+ 1	− 1	72	74	72
4	+ 1	+ 1	− 1	100	100	100
5	− 1	− 1	+ 1	37	36	37
6	+ 1	− 1	+ 1	43	41	45
7	− 1	+ 1	+ 1	73	75	72
8	+ 1	+ 1	+ 1	96	97	95

**Fig. 9:**
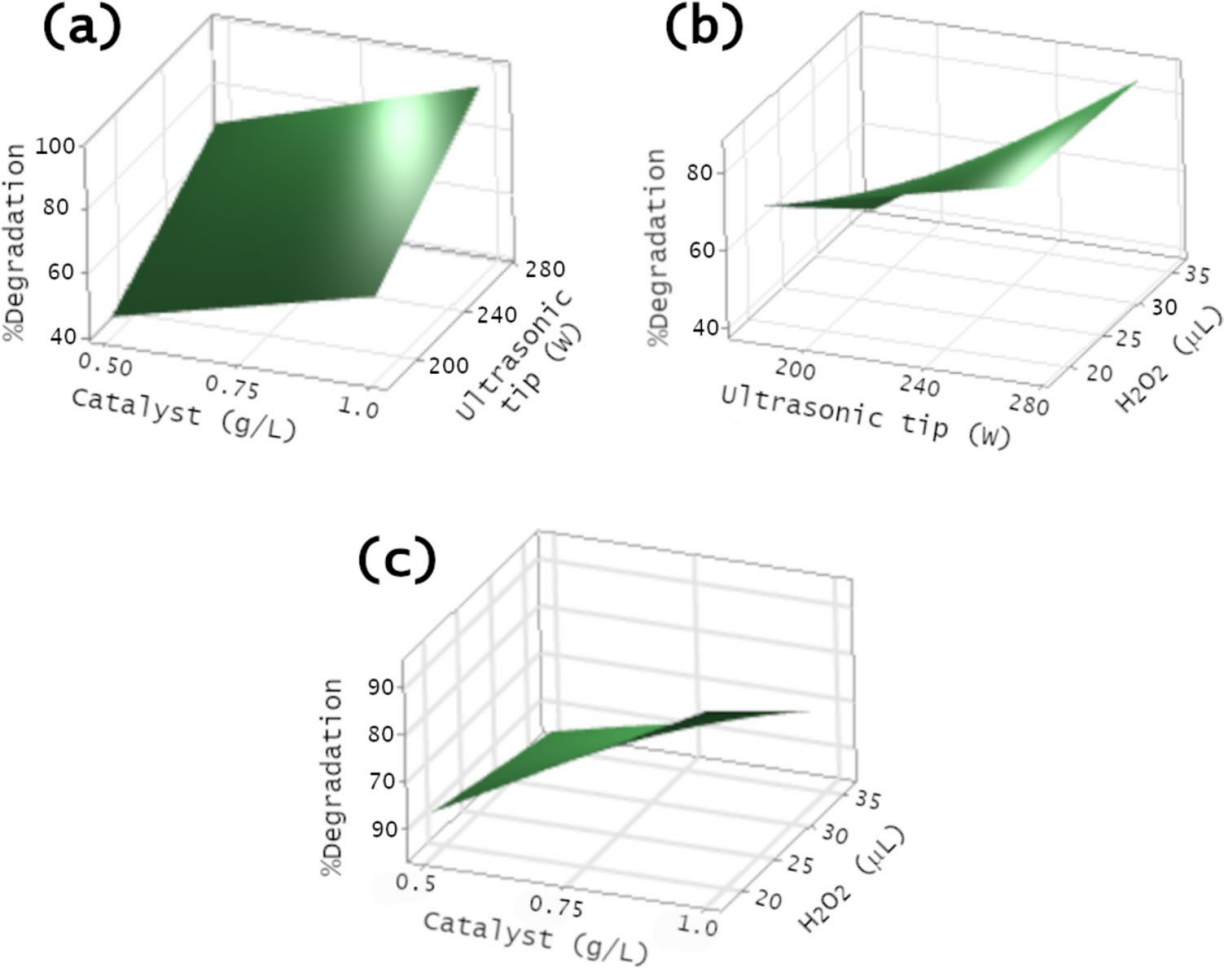
Contour plot of sonocatalytic degradation of RB-5 as a function of catalyst dosage, ultrasonic probe power, and hydrogen peroxide, employing 1.0%W-ZnO as catalyst

The response surface and contour plots for dye degradation were obtained by using the Minitab software. The cube plot visually represents the impact of catalyst dosage, ultrasonic power tip, and hydrogen peroxide volume on RB-5 sonocatalytic degradation, as depicted in Fig. S7; see SI. It is noteworthy that the power tip where a higher degradation was observed, specifically 270 W, is the one used as the maximum value in the factorial design. According to the cube plot, optimal conditions for RB-5 degradation include a catalyst dosage of 1.0 g/L, a power tip of 270 W, and a hydrogen peroxide volume of 18 μL. On the contrary, the least favorable conditions for dye degradation were a catalyst dosage of 0.5 g/L, a power tip of 180 W, and a peroxide volume of 36 μL. Analogously, the surface diagrams in Fig. 9 provide a clearer depiction of how catalyst dosage, ultrasonic tip power, and hydrogen peroxide volume impact dye degradation. In Fig. 9(a), degradation is plotted against catalyst concentration and ultrasonic tip power, revealing that degradation benefits from higher catalyst concentration and power at the tip. Conversely, Fig. 9(b) illustrates degradation concerning hydrogen peroxide and ultrasonic power tip, showing that the greatest degradation occurs with the lowest amount of hydrogen peroxide and the highest power in the ultrasonic tip. Finally, Fig. 9(c) displays dye degradation as a function of catalyst and hydrogen peroxide concentration. According to these results, higher peroxide concentrations result in lower degradation percentages, and higher catalyst concentrations correlate with increased sonocatalytic activity.

Finally, the degradation of the RB-5 azo dye is favored by the use of W-doped ZnO as a catalyst in conjunction with the ultrasonic power tip. Additionally, it is evident that a higher volume of hydrogen peroxide restrains the degradation of RB-5. Moreover, based on the results obtained from the 2^*k*^ factorial design, the optimal conditions found for a better degradation of RB-5 consider a catalyst dosage of 0.75 g/L, a power setting at the ultrasonic tip of 225 W, and a hydrogen peroxide volume of 27 μL. To validate these findings, the degradation of the RB-5 dye was carried out at 20 ppm concentration under the best conditions determined by the factorial design. The sonocatalytic activity of 1.0%W-ZnO under the best conditions found by the factorial design leads to a total dye degradation within 60 min. This performance is similar to the degradation achieved under conditions involving an ultrasonic power tip of 270 W, a catalyst concentration of 1 g/L, and a hydrogen peroxide volume of 18 μL, where complete degradation was observed within 80 min; see Fig. S8 from SI. These results confirm the efficacy of the factorial design in determining optimal conditions within a specified parameter interval to perform dye degradation. In addition, according to other authors, this degradation method helps not only to degrade highly stable organic contaminants but also to obtain high levels of mineralization (Abdelhaleem and Chu 2017, 2018).

## Conclusions

Doping ZnO nanoparticles with rhodium and tungsten at 1.0% does not alter the wurtzite hexagonal structure of the material, but it does induce slight modifications in the cell parameters. This alteration is attributed to differences in electronic density, leading to an increase in cell parameters when doped with rhodium and a decrease with tungsten doping. In addition, the insertion of rhodium into the ZnO lattice causes a higher degree of distortion, resulting in a subtle blue shift. Furthermore, the incorporation of dopants contributes to a more effective separation of charge carriers, enhancing the sonocatalytic activity of ZnO. The doped and co-doped samples also exhibit a higher presence of hydroxyl groups, which, combined with oxygen vacancies, favors sonocatalytic activity, particularly in the case of W-ZnO. Interestingly, contrary to initial assumptions, co-doping ZnO leads to a decrease RB-5 degradation compared to the doped counterpart. Besides, it was found that high concentrations of hydrogen peroxide unfavorably impact the sonocatalytic activity of RB-5 degradation, as revealed in the 2^*k*^ factorial design, where the optimal degradation conditions found were a catalyst concentration of 0.75 g/L, a power tip of 225 W, and a hydrogen peroxide volume of 27 μL.

## Supplementary Information

Below is the link to the electronic supplementary material.

Supplementary file1 (DOCX 2889 KB)

## Data Availability

The data supporting the findings of this study are available from the corresponding author upon reasonable request.
