# Supplementary material for: Sonocatalytic degradation of RB-5 dye using ZnO nanoparticles doped with transition metals
Source: Environ Sci Pollut Res Int. 2024 Dec 20;32(2):783–97. doi: 10.1007/s11356-024-35776-4 (PMC11732947; doi:10.1007/s11356-024-35776-4)
Supplement: Supplementary file 1 — Supplementary file1 (DOCX 2889 KB) [file 11356_2024_35776_MOESM1_ESM.docx]

**Sonocatalytic degradation of RB-5 dye using ZnO nanoparticles doped with transition metals.**

Tatiana Rodríguez-Flores^a^, Isaías Hernández-Pérez*^a^, Gloria Elena de la Huerta-Hernández^a^, Raúl Suárez-Parra^b^, Catalina Haro-Pérez ^a^

^a^ Departamento de Ciencias Básicas, Universidad Autónoma Metropolitana-Azcapotzalco, Av. San Pablo 420, C.P 02128, Ciudad de México, México

^b^ Departamento de Materiales Solares, Instituto de Energías Renovables, Universidad Nacional Autónoma de México, Priv. Xochicalco s/n, C.P. 62580 Temixco, Morelos, México

*Corresponding Author: [ihp@azc.uam.mx](mailto:ihp@azc.uam.mx);


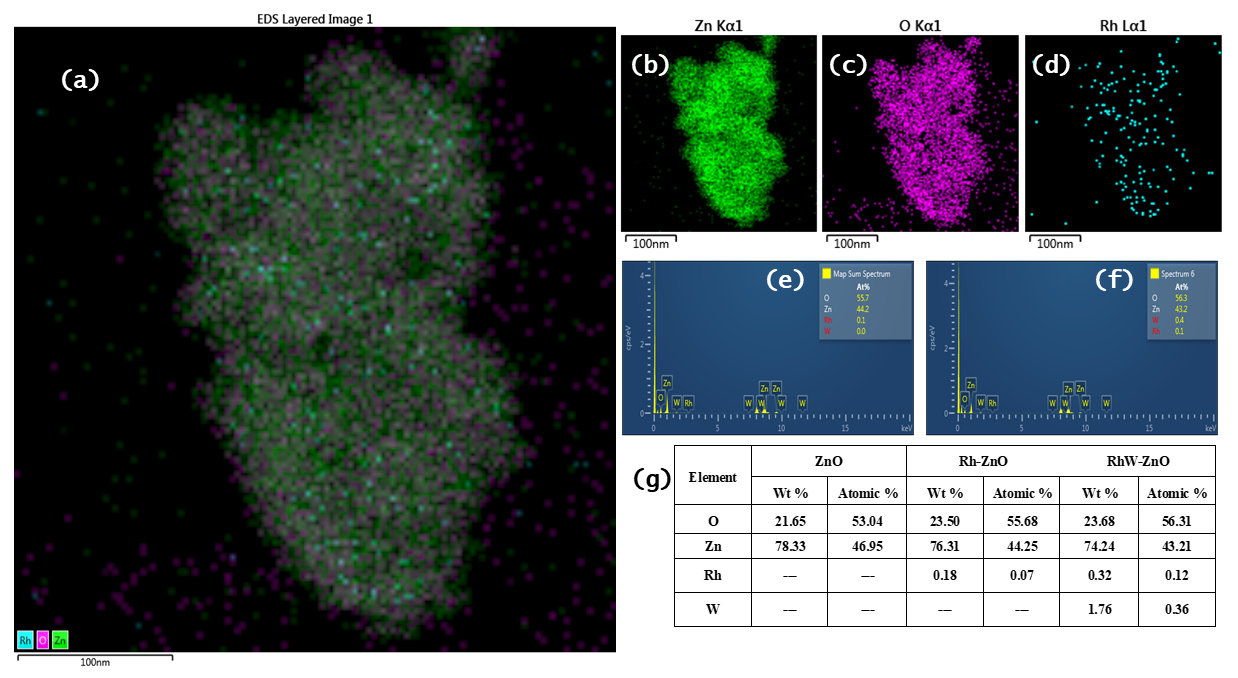


Fig. S1. HRTEM analysis: (a) Rh-ZnO sample, (b), (c), and (d) show the EDS mapping of Zn, O and Rh distribution, respectively, of the Rh-ZnO sample, (e) Spectrum of Rh-ZnO, (f) Spectrum of RhW-ZnO, and (g) Elemental composition of pure ZnO, Rh-ZnO, and RhW-ZnO.

Scherrer equation

|  | $\text{D =}\frac{\text{kλ}}{\text{β(cosθ)}}$ | Eq. (S1) |
| --- | --- | --- |

where k is a constant (0.9), λ is the X-ray wavelength, β is the full-width at half maximum of the diffraction peak, and θ is the Bragg angle.

Bragg’s law principles for hexagonal wurtzite structure

|  | $\frac{\text{1}}{\text{d}^{\text{2}}}\text{ =}\frac{\text{4}}{\text{3}}\left( \frac{\text{h}^{\text{2}}\text{+hk+}\text{k}^{\text{2}}}{\text{a}^{\text{2}}} \right)\text{+}\frac{\text{l}^{\text{2}}}{\text{c}^{\text{2}}}$ | Eq. (S2) |
| --- | --- | --- |

Where *d* is the interplanar distance, *h*, *k*, and *l* are the Miller indices of the crystal planes and *a* and *c* are the lattice constants. From this equation and the X-ray diffraction patterns, the lattice parameters of pure and doped ZnO were calculated.

The XRD patterns were refined using a linear interpolation between a set of background points with the Thompson-Cox-Hasting function, considering anisotropic samples. The refinement parameters included instrumental parameters, lattice parameters, asymmetries, atomic positions, and shape parameters. This approach aimed to determine the occupancy of pure and doped ZnO and to confirm the presence of dopants in the crystal structure. The occupancies were calculated from dopant/multiplicity ratio.

Table S1. Theoretical and refined occupancies of pure, doped and co-doped ZnO with Rh and W.

| Element | Theorical occupancies | | | | Refined occupancies | | | |
| --- | --- | --- | --- | --- | --- | --- | --- | --- |
|  | ZnO | Rh-ZnO | W-ZnO | RhW-ZnO | ZnO | Rh-ZnO | W-ZnO | RhW-ZnO |
| Zn | 0.04167 | 0.04125 | 0.04125 | 0.04125 | 0.04160 | 0.04124 | 0.04592 | 0.04516 |
| O | 0.04167 | 0.04167 | 0.04167 | 0.04167 | 0.04160 | 0.04160 | 0.05925 | 0.06064 |
| Rh | -- | 0.00042 | -- | 0.00021 | -- | 0.00041 | -- | 0.00412 |
| W | -- | -- | 0.00042 | 0.00021 | -- | -- | 0.00509 | 0.00413 |


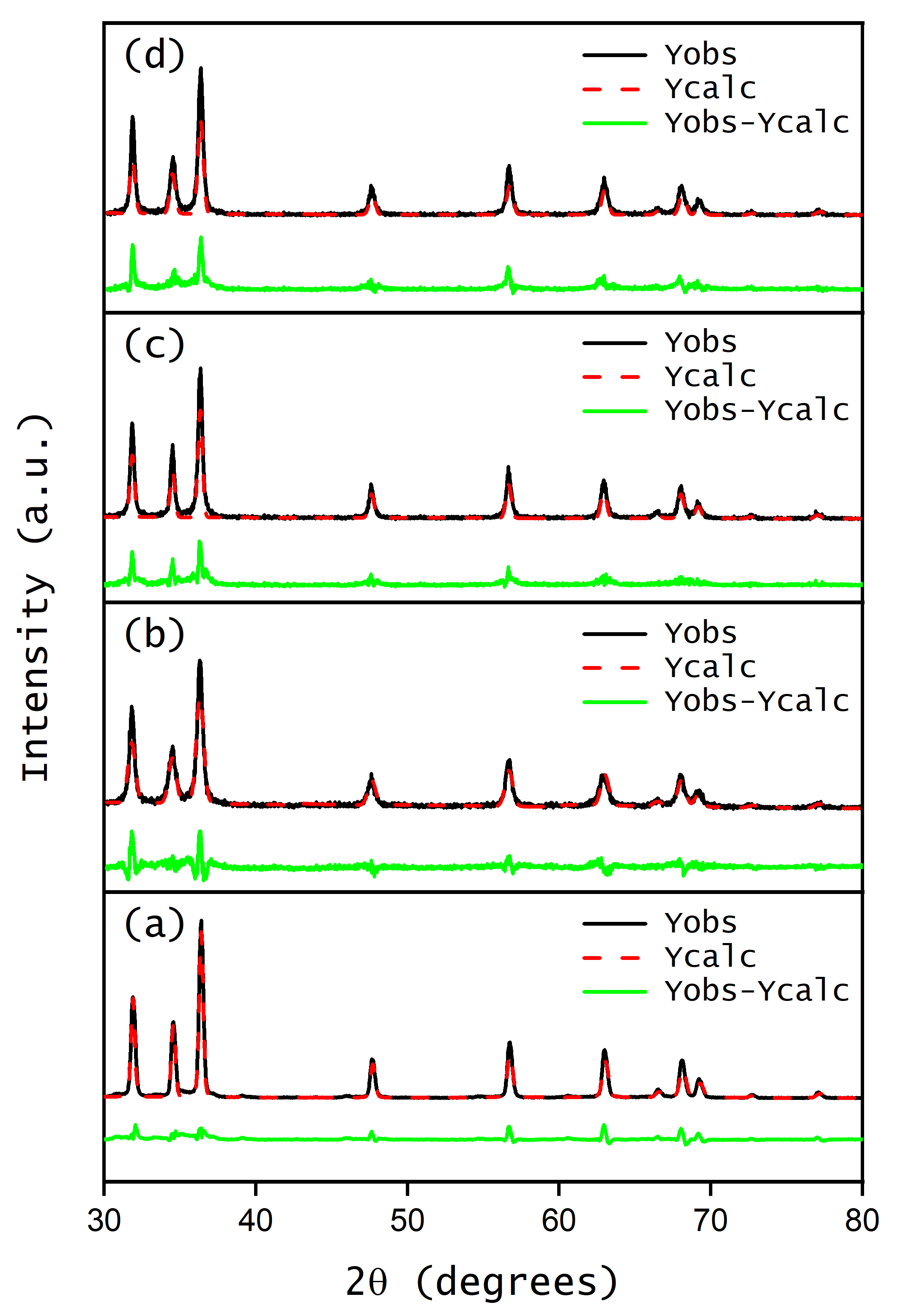


**Fig. S2.** Rietveld refinement plot of pure, doped and co-doped ZnO with rhodium and tungsten; (a) pure ZnO (b) Rh-ZnO, (c) W-ZnO, and (d) RhW-ZnO.

The band gap was obtained using the data from diffuse reflectance and the Kubelka-Munk transformation, and Tauc equation, equation (S3) and equation (S4), respectively:

|  | $\text{E=}\frac{\text{hc}}{\text{λ}}$ | Eq. (S3) |
| --- | --- | --- |
|  | $\left( \text{αhν} \right)^{\text{2}}\text{=A}\left( \text{hν-}\text{E}_{\text{g}} \right)$ | Eq. (S4) |

Therefore, the final equation for the Kubelka-Munk transformation is obtained by considering the relationship between diffuse reflectance and absorption α and it is expressed as follows:

|  | $\text{E=}\left( \text{F(R)×hν} \right)^{\text{2}}$ | Eq. (S5) |
| --- | --- | --- |

Where h is Planck's constant, ν is the frequency. Therefore, the band gap will be determined by the linear intersection on the x-axis of the (F(R) x hν)² vs Energy plot, when F(R) = 0.

The Urbach energy was calculated from the F(R) results according to the following equation:

|  | $\text{α=}\text{α}_{\text{0}}\text{e}^{\left( \frac{\text{hν}}{\text{E}_{\text{u}}} \right)}$ | Eq. (S6) |
| --- | --- | --- |

where $\text{α}_{\text{0}}$ is a constant, $h\nu$ is the photon energy, $\text{E}_{\text{u}}\text{ }$is the Urbach energy, and $\text{α}$ is the absorption coefficient. Hence, the Urbach energy was obtained from the ln(F(R)) vs hν plot, where E_u_ is equal to the inverse of the slope.


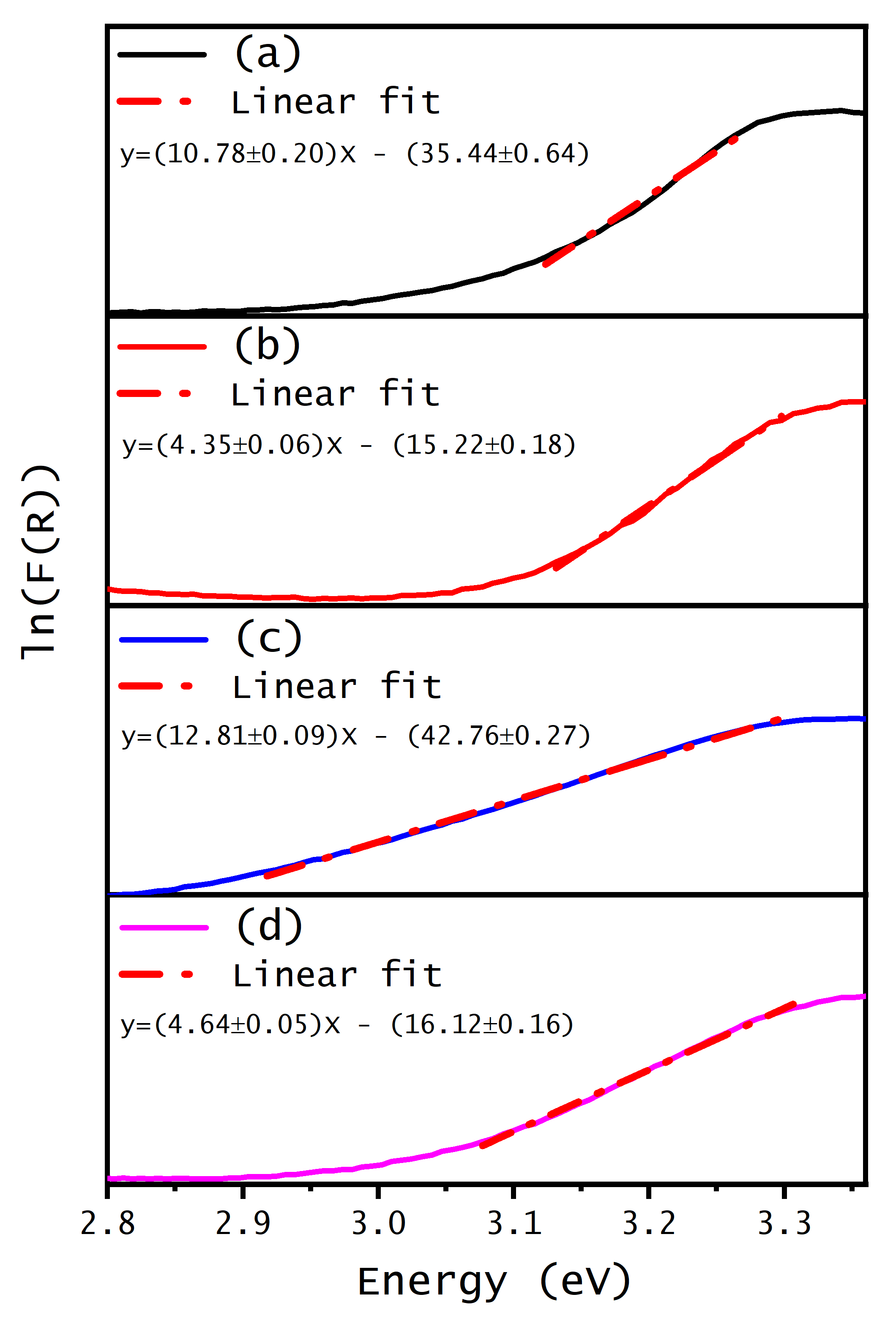


**Fig. S3** Urbach energy of (a) undoped ZnO, (b) 1.0%Rh-ZnO, (c) 1.0%W-ZnO, and (d) 1.0%RhW-ZnO.


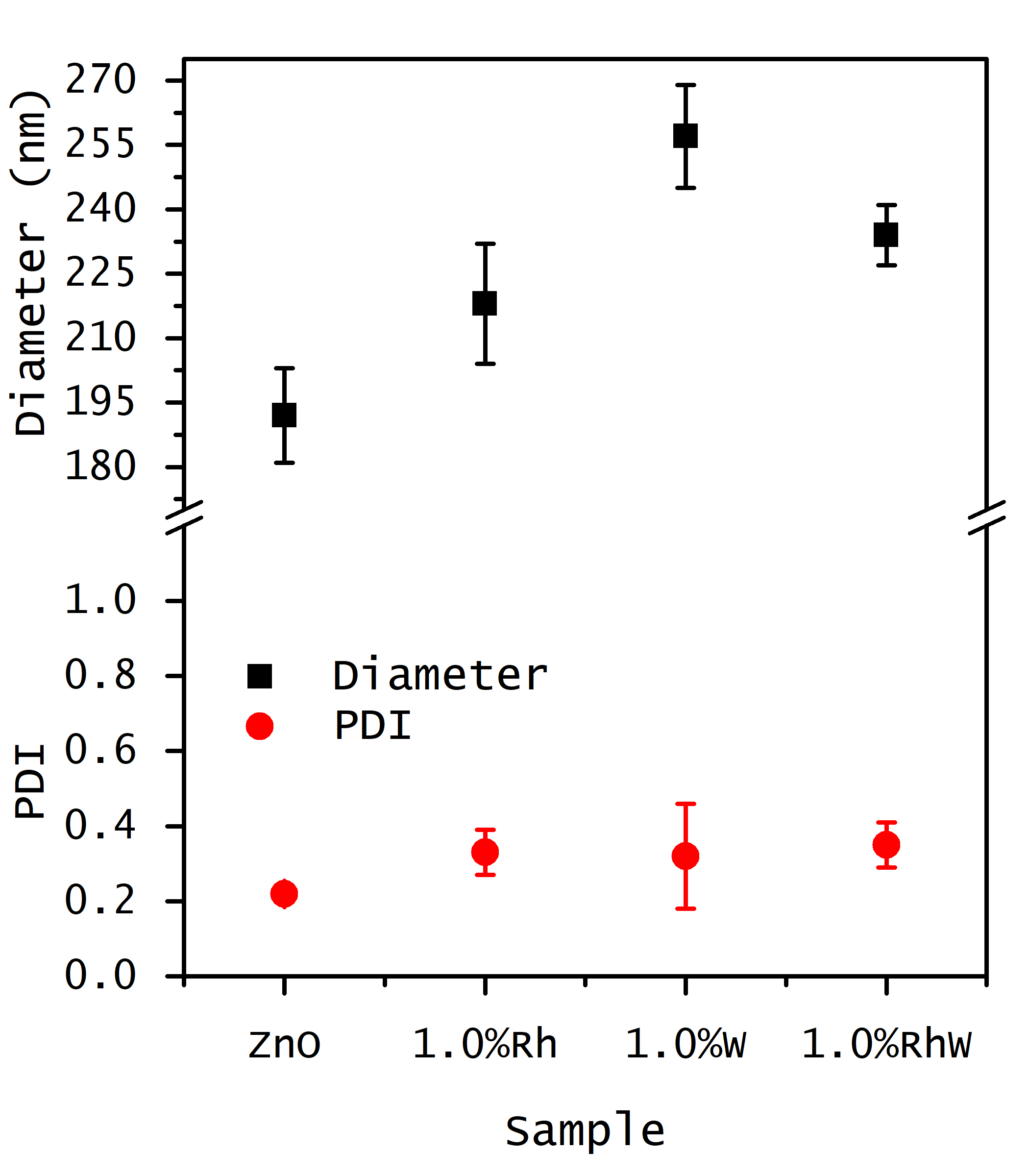


**Fig. S4** Hydrodynamic diameter and polydispersity index of undoped ZnO and 1.0% doped and co-doped ZnO with rhodium and tungsten measured by dynamic light scattering


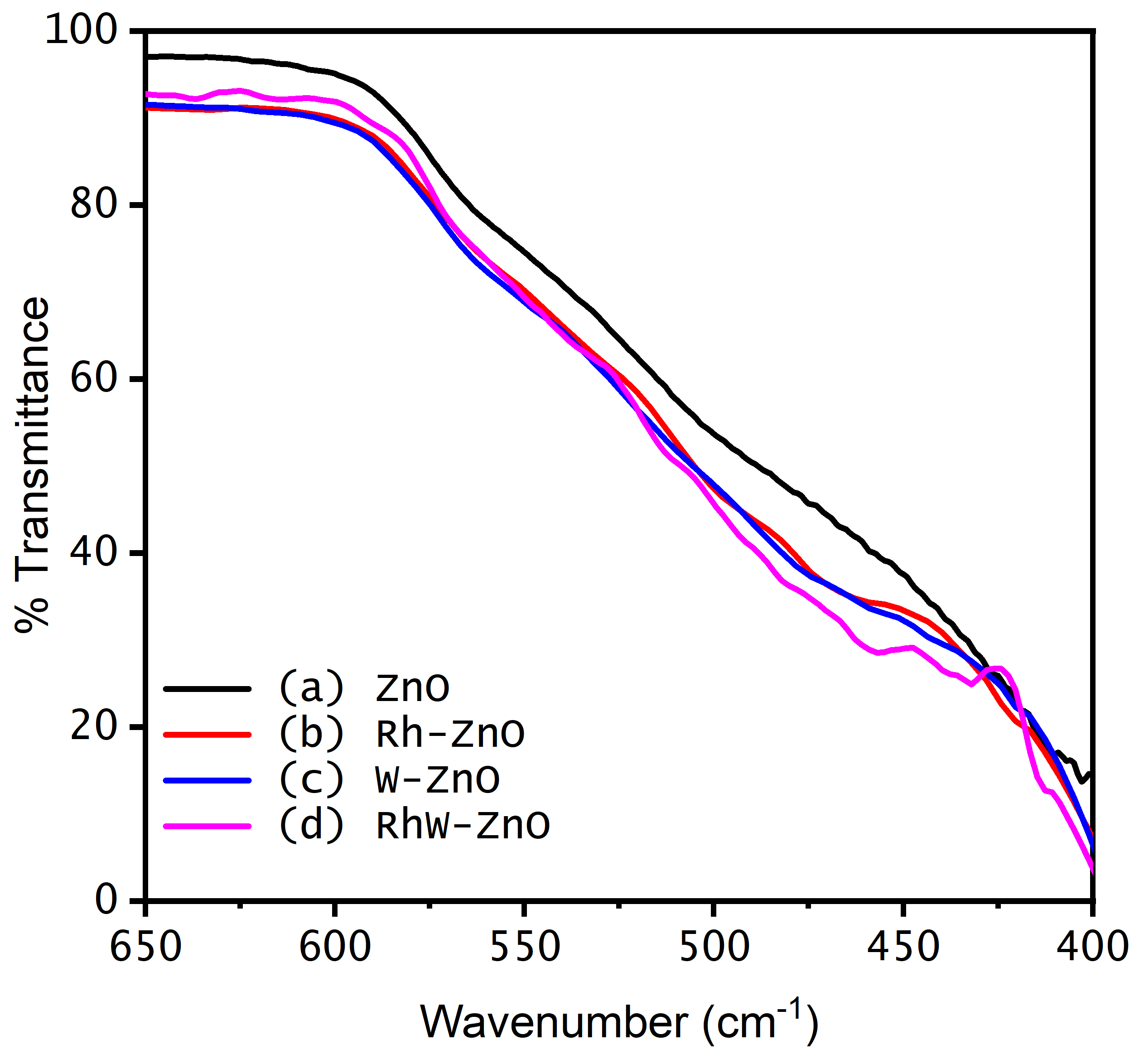


**Fig. S5** Zoomed region of the Fourier transform infrared spectra from 650 to 400 cm⁻¹ for pure, doped, and co-doped ZnO samples: (a) ZnO, (b) Rh-ZnO, (c) W-ZnO, and (d) RhW-ZnO.


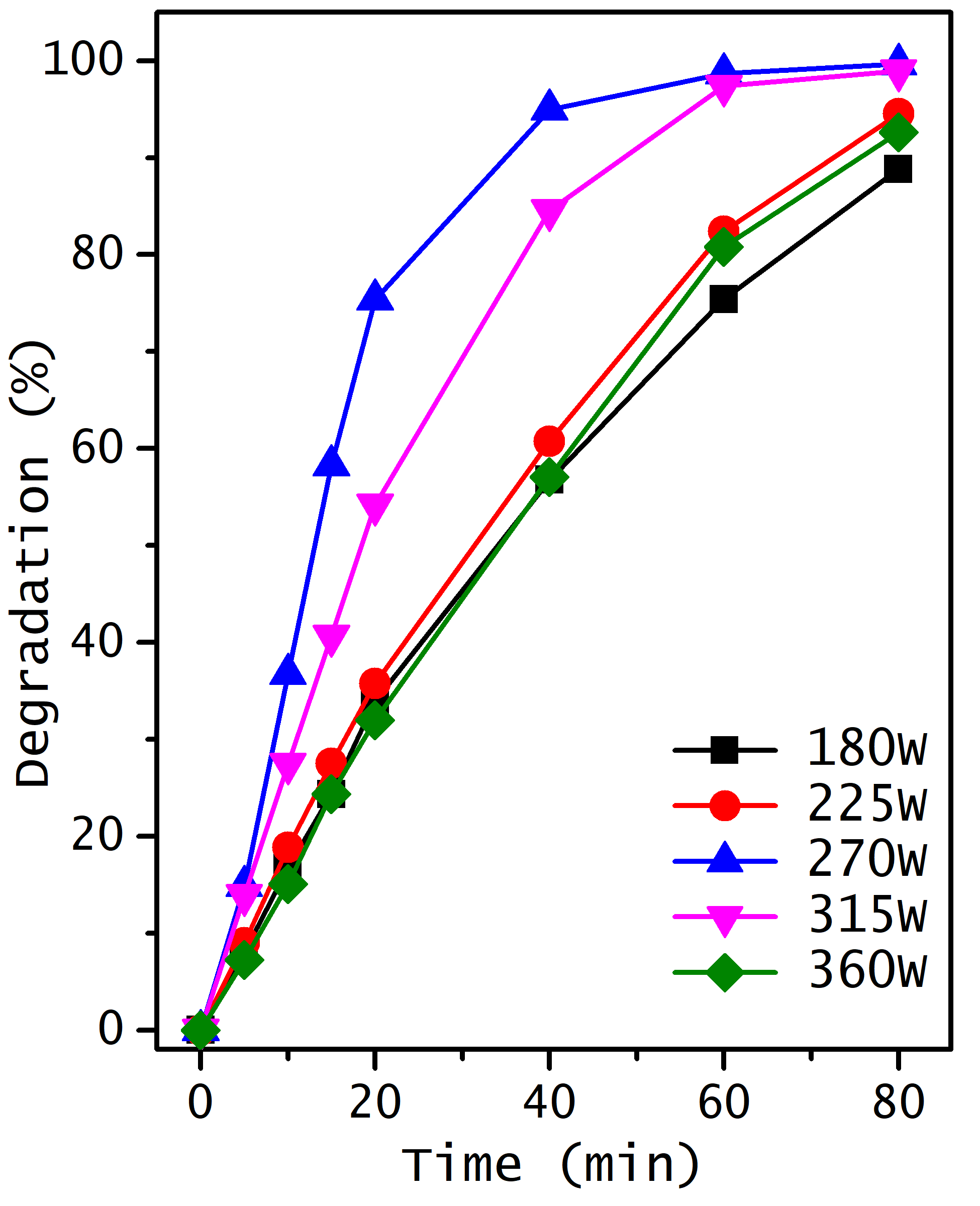


**Fig. S6** Degradation percentage of the RB-5 reactive azo dye by sonocatalysis using ZnO doped with 1.0% W, varying the power from 180 watts to 360 watts, with a catalyst dosage of 1.0 g/L, and concentration of 6x10^-3^M of hydrogen peroxide.


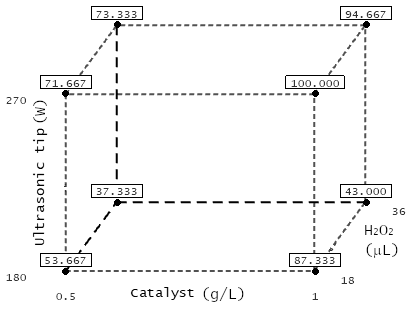


***Fig. S7*** *Cubic plot illustrating the sonocatalytic degradation percentage of dye using W-doped ZnO as a catalyst obtained for various concentrations of the catalyst, hydrogen peroxide, and ultrasonic tip power*


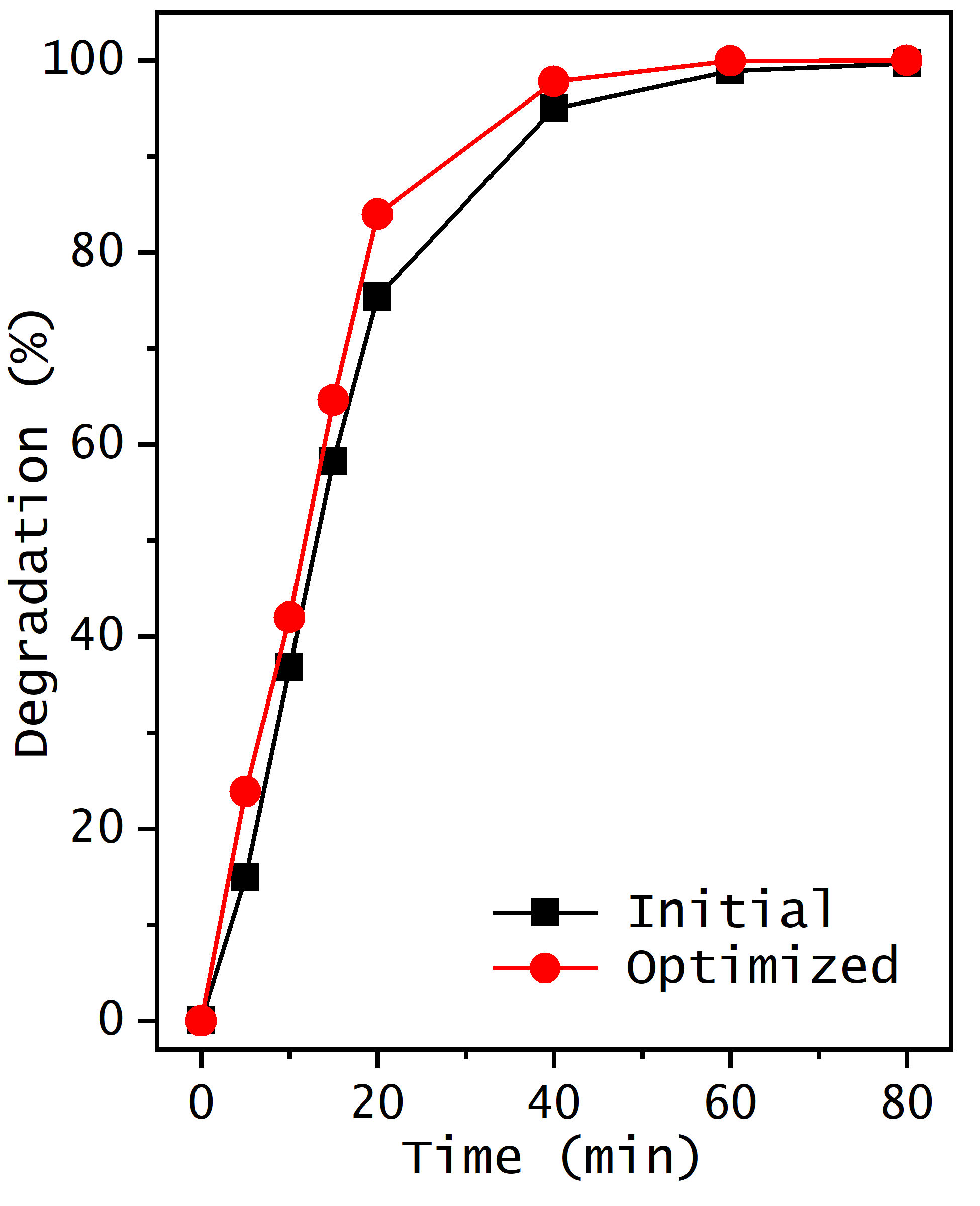


**Fig. S8** Time evolution of the RB-5 dye degradation reaction through sonocatalysis employing 1.0% W-ZnO as the catalyst, under the optimized conditions identified by the factorial design (red symbols) and the proposed initial conditions (black symbols). The optimal conditions are a catalyst dosage of 0.75 g/L, a power tip of 225 W, and a hydrogen peroxide volume of 27 μL. The proposed initial conditions are a catalyst dosage of 1.0 g/L, a power tip of 270 W, and a hydrogen peroxide volume of 18 μL.
